# The gene regulatory molecule GLIS3 in gastric cancer as a prognostic marker and be involved in the immune infiltration mechanism

**DOI:** 10.3389/fonc.2023.1091733

**Published:** 2023-02-27

**Authors:** Yi Ding, Zehua Wang, Chen Chen, Chenxu Wang, Dongyu Li, Yanru Qin

**Affiliations:** ^1^ Department of Oncology, The First Affiliated Hospital of Zhengzhou University, Zhengzhou, China; ^2^ School of Pharmacy, Macau University of Science and Technology, Macao, Macao SAR, China

**Keywords:** GLIS3, prognosis, biomarker, gastric cancer, immune infiltration

## Abstract

**Background:**

Gastric cancer is the most prevalent solid tumor form. Even after standard treatment, recurrence and malignant progression are nearly unavoidable in some cases of stomach cancer. GLIS Family Zinc Finger 3 (GLIS3) has received scant attention in gastric cancer research. Therefore, we sought to examine the prognostic significance of GLIS3 and its association with immune infiltration in gastric cancer.

**Method:**

Using public data from The Cancer Genome Atlas (TCGA), we investigated whether GLIS3 gene expression was linked with prognosis in patients with stomach cancer (STAD). The following analyses were performed: functional enrichment analysis (GSEA), quantitative real-time PCR, immune infiltration analysis, immunological checkpoint analysis, and clinicopathological analysis. We performed functional validation of GLIS3 *in vitro* by plate cloning and CCK8 assay. Using univariate and multivariate Cox regression analyses, independent prognostic variables were identified. Additionally, a nomogram model was built. The link between OS and subgroup with GLIS3 expression was estimated using Kaplan-Meier survival analysis. Gene set enrichment analysis utilized the TCGA dataset.

**Result:**

GLIS3 was significantly upregulated in STAD. An examination of functional enrichment revealed that GLIS3 is related to immunological responses. The majority of immune cells and immunological checkpoints had a positive correlation with GLIS3 expression. According to a Kaplan-Meier analysis, greater GLIS3 expression was related to adverse outcomes in STAD. GLIS3 was an independent predictive factor in STAD patients, as determined by Cox regression (HR = 1.478, 95%CI = 1.478 (1.062-2.055), P=0.02)

**Conclusion:**

GLIS3 is considered a novel STAD patient predictive biomarker. In addition, our research identifies possible genetic regulatory loci in the therapy of STAD.

## Introduction

Globally, gastric cancer (GC) is the fourth highest cause of cancer-related mortality (1). Most GC patients have a dismal prognosis due to late diagnosis and inadequate response to existing therapy. Despite continued advances in treatment, GC mortality remains high. Approximately 50% of patients with advanced GC experience recurrence after the first curative resection, The prognosis for patients with progressive GC that is recurrent or unresectable remains dismal, with a median survival time of fewer than 12 months with current standard treatment (2). Therefore, we explored prognostic genetic biomarkers to predict patient survival and response to individualized therapy.

Biomarkers are specific indicators of normal biological, pathogenic, or pharmacological responses to therapeutic interventions. They are features that are objectively measured and evaluated. Effective biomarker screening, it is possible to detect GC earlier and reduce GC mortality. Biomarkers can be produced directly by cancer cells or non-cancerous cells responding to the tumor. The biomarkers found in gastric cancer today are broadly classified into three categories, immune, molecular, and genetic related. Carcinoembryonic antigen (CEA) is gastric cancer most common tumor marker in gastric cancer (3). CA125, CA19-9, CA72-4, and alpha-fetoprotein have also been reported to contribute to the prognosis of gastric cancer (4–6). Furthermore, tumor markers associated with invasion and metastasis and extracellular matrix (ECM) adhesion and degradation continue to play a role in cancer prognosis. Upregulation or alteration of ECM molecules usually indicates the malignant progression of cancer cells. These include proteases, calmodulin, mucin, and CD44 splice variants (7–9). While genetic changes include genetic instability represented by microsatellite instability (10), reactivation of telomerase activity, inactivation of tumor suppressor genes, and activation of oncogenes (11). Current biomarkers commonly used for clinical testing are CA19-9, CEA, CA72-,4, and PG I/II. The sensitivity and specificity of present biomarker tests still need to be improved and not the best choice for screening GC. Studies based on multiple biomarker assays only help to monitor prognostic indicators in gastric cancer patients after treatment (12). Therefore, further studies on biomarkers are necessary.

GLI-Similar 3 (GLIS3) is a member of the GLIS subfamily of Krüppel-like zinc-finger transcription factors that regulate gene expression (13, 14). GLIS3 is essential for controlling numerous physiological processes and has been linked multiple diseases, including neonatal diabetes, glaucoma, polycystic kidney disease, neurological disorders, congenital hypothyroidism, and cancer (15–18). The expression pattern of GLIS3 varies significantly in different types of cancers. GLIS3 is detected in the highly proliferative group of central neurological tumors such as ventricular meningioma and cerebral glioblastoma (19, 20). In contrast, reduced GLIS3 expression was observed in chromophobe renal cell carcinoma (21). However, any association of GLIS3 with gastric cancer has hardly been carefully studied.

These data were obtained from TCGA. We analyzed the pattern of GLIS3 expression in gastric cancer and its predictive value. A high GLIS3 level predicted a poor prognosis for people with GC. In addition, GLIS3 is related to immunological response, which offers a novel perspective for tailored therapy. According to this article, high GLIS3 expression is related to poor outcomes in GC patients, and that GLIS3 helps to predict the prognosis of GC patients.

## Methods

### Patient data sets

We universally processed RNAseq data in TPM format for TCGA, GEO database GSE62254 using UCSC XENA (https://xenabrowser.net/datapages/ ) *via* Toil (22). STAD (gastric cancer) data from TCGA. In addition, the mRNA expression data (407 samples, process type: HTSeq-FPKM) and clinical information were extracted from the TCGA database (https://cancergenome.nih.gov). This work follow TCGA publication criteria to the letter.

### Quantitative reverse transcription PCR

Quantitative real-time polymerase chain reaction (qRT-PCR) RNA samples were obtained from 10 pairs of primary adenocarcinoma tissues and paraneoplastic tissues provided by Linzhou Cancer Hospital (Henan, China). All participants provided written informed permission for this study, which was authorized by the First Affiliated Hospital of Zhengzhou University’s institutional ethics. Following the manufacturer’s directions, total RNA was extracted using a TRIzol reagent (Servicebio, Wuhan, China). A cDNA synthesis kit (Servicebio, Wuhan, China) was used to reverse-transcribe identical quantities of RNA (1 μg). Complementary DNAs (cDNAs) were analyzed by qPCR using SYBR Green Supermix reagent (Thermo Fisher, America) at a final dilution of 1:5. Using GAPDH as a reference gene. The following primers were used in this study: GLIS3 F, TTACAGAGGGCAATGAATGCAG; R, AGACTCACGCGAAATAAGGGA; GAPDH F, CAGGAGGCATTGCTGATGAT; R, GAA GGCTGGGCTCATTT.

### Western blot

Total protein was extracted from grown cells using RIPA buffer (epizme, Shanghai, China) containing protease and phosphatase inhibitors, and total protein was determined using a BCA protein assay kit (Thermo Fisher, USA). Protein samples were separated using 10% SDS-PAGE, transferred to polyvinylidene difluoride (PVDF) membranes (Millipore, Billerica, MA), and incubated with primary and secondary antibodies. Protein bands were identified by a protein imaging system (Amersham Imager 600).

### Cell counting kit-8 assays

CCK-8 assays were performed in 96-well plates at a cell density of 1*10^3^ cells/well, providing 200 µl of medium (10%FBS and RPMI-1640 culture medium) per well. After the prescribed time (every 24H), CCK-8 reagent and 100 μl of media were added to each well, and cells were incubated at 37°C for 2 hours. The absorbance at 490 nm was measured using an enzyme marker to compute the cell growth rate.

### Colony formation assay

After inoculation of 1000 cells per well in a 6-well plate, cell culture was performed for one week. 4% paraformaldehyde was used to fix the cells for 30 minutes, and 1% crystalline violet staining solution was used to stain them for 30 minutes at room temperature. These plates were air-dried and thoroughly washed before being photographed.

### Wound healing assay

Gastric cancer cells were seeded in 6-well plates. After the cells grew to 100% fusion, the cell layer was scratched with the tip of a 200 µl pipette, and the medium containing 10% fetal bovine serum was replaced with a serum-free medium. Images of the cells were captured at 0 and 48, respectively.

### Cell migration assay

In migration assays, 5x10^4^ gastric cancer cells were inoculated into Transwell chambers in serum-free medium; the chambers were inserted above a 24-well plate containing 20% FBS medium. After incubation at 37˚C with 5% CO2 for 24 hours, the Transwell chamber was removed, and the medium in the smaller chamber was discarded and washed with PBS. The cells were then fixed with 4% paraformaldehyde for 30 min and stained with 0.1% crystal violet for 30 min. The top unmigrated cells were gently swabbed off with a cotton swab and observed and photographed under a microscope.

### Differential expression gene analysis

The median GLIS3 expression was used as the cut-off value (HTseq-Count) to distinguish between low and high GLIS3 expression in STAD samples. And Differential expression gene (DEG) analysis was performed using the DESeq2 R package (1.26.0) (23, 24).

### Functional enrichment analysis

The DEGS threshold for functional enrichment analysis was defined as logFC greater than two and adjusted P value less than 0.05 for upregulated gene sets. Gene ontology (GO), including biological process (BP), cellular component (CC) and molecular function (MF), and Kyoto Encyclopedia of Genes and Genomes (KEGG) analysis using the clusterProfiler package (version 3.14.3 version) (for enrichment analysis); org.Hs.eg.db package (version 3.10.0) (for ID conversion).

### Gene set enrichment analysis

GSEA is a computational tool for determining if a previously defined set of genes demonstrates statistically and persistently significant differences between two biological states (25). We utilized the ClusteProfile R Package (3.14.3) to investigate functional and route differences between the two groups with distinct GLIS3 expressions. The number of permutations for each analysis was set to 1000. Significant enrichment was determined to exist when the False discovery rate (FDR) was less than 0.25, and p.adjust was less than 0.05 (26).

### Immunoassay

Using the GSVA R package (1.34.0), we performed a single sample gene set enrichment analysis (ssGSEA) for the immune infiltration study of GLIS3 (27). Twenty-four distinct types of invading immune cells were analyzed (28). The link between GLIS3 and immunological checkpoints, including PD1, PD-L1, CTLA4, LAG3, TIGIT, and CD48, was then investigated further (ggplot2 3.3.3).

### Statistical analyses

Using the R programming language, all statistical analyses and visualizations were generated (version 3.6.3). Wilcoxon rank-sum test was used to evaluate the expression of GLIS3 in samples that were not paired. The diagnostic value of GLIS3 gene expression was determined using ROC curves, with the area under the ROC curve serving as the diagnostic value. Univariate COX analysis was performed to screen for potential prognostic markers, and multivariate COX analysis was used to confirm the influence of GLIS3 expression on survival in conjunction with other clinical variables. Combining GLIS3 expression with clinical factors, a nomogram was developed to predict STAD patients’ overall survival at 1, 3, and 5 years. Utilizing Kaplan-Meier survival analysis, the survival distribution was estimated. P-values below 0.05 were considered statistically significant.

## Results

### High expression of GLIS3 in gastric cancer

**Figure 1:**
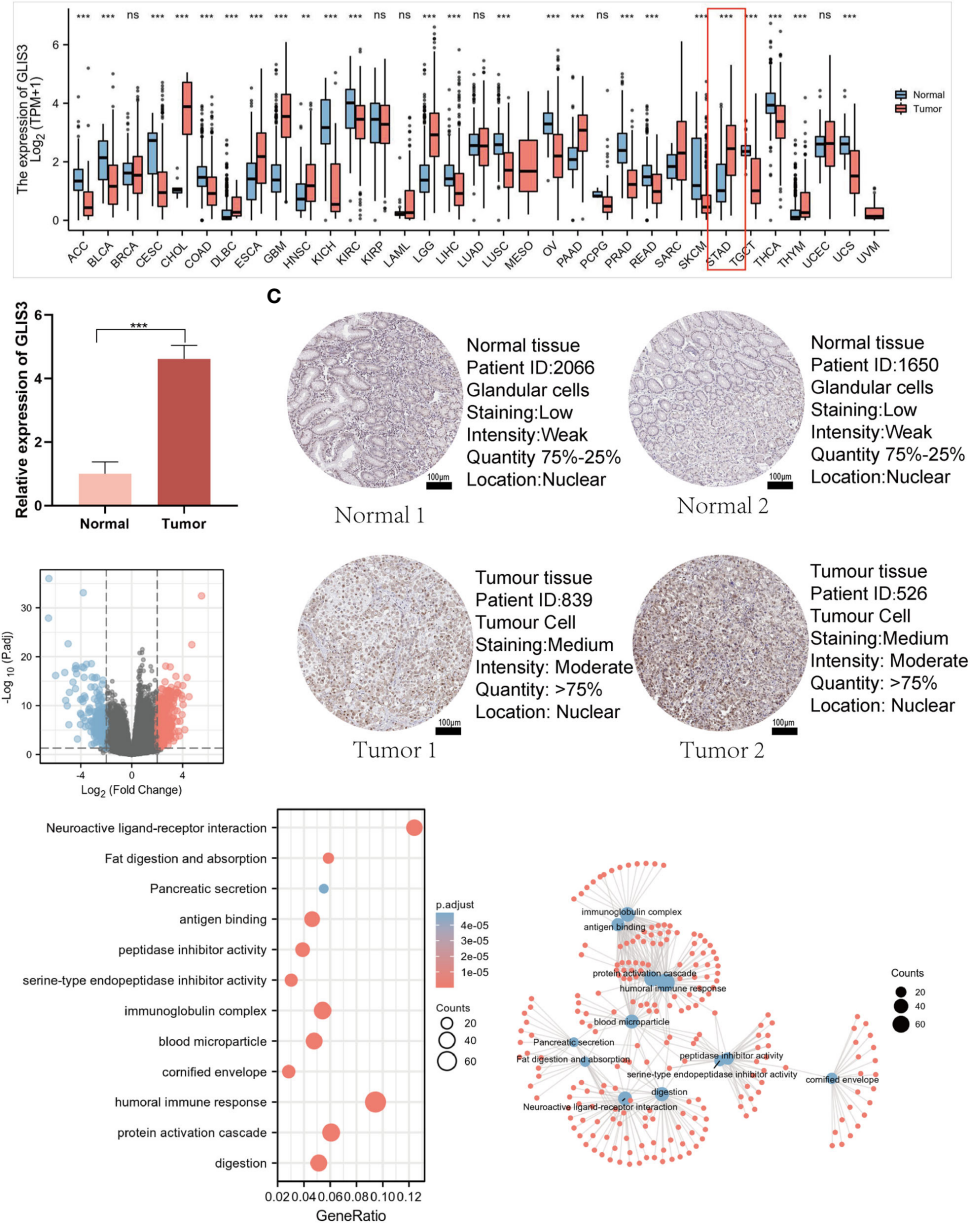
GLIS3 expression and functional analysis. **(A)** Elevated or decreased GLIS3 in cancer and paracancerous tissues in different tumor types from The Cancer Genome Atlas (TCGA) database. **(B)** qRT-PCR for the detection of GLIS3 gene expression in 10 cases of paracancerous tissues and gastric cancer tissues. **(C)** GLIS3 immunohistochemical staining of gastric cancer and normal gastric tissues in the HPA database. (Scale Bar=100μm) **(D)** A total of 354 up-regulated genes and 252 down-regulated genes were identified as statistically significant in the GLIS3 high and low expression groups. Where red dots indicate upregulated genes, blue dots imply downregulated genes, and gray is not statistically significant. **(E)** GO enrichment analysis and connection diagram with a visual network; BP, biological process; CC, cellular composition; MF, molecular function. (***P <0.001, ns, No sense).

Comparing GLIS3 expression in normal tissues and tumor samples from the TCGA and GTEx databases, we discovered that GLIS3 expression differed significantly in the majority of cancer types (**Figure 1A**). We verified the expression of GLIS3 mRNA in gastric cancer tissues by quantitative qRT-PCR. We found that GLIS3 mRNA expression was upregulated in gastric cancer tissues (N=10) compared with normal gastric tissues (P<0.001, **Figure 1B**). In addition, GLIS3-related protein expression data are available in the HPA database. Immunohistochemical results showed that GLIS3 expression was higher in gastric cancer compared to normal tissues. (**Figure 1C**).

### Identification of DEGs with GLIS3 and functional enrichment analysis

DEG identification with GLIS3 was performed using |logFC| >2 and PADJ <0.05. A total of 606 DEGs comprised 354 up-regulated genes, and 252 down-regulated genes were discovered between the two groups of low and high GLIS3 expression (**Figure 1D**). The following are the outcomes of GO functional analysis and KEGG enrichment analysis. BP includes a humoral immune response, protein activation cascade, and digestion. CC consisted of an immunoglobulin complex, blood microparticle, and cornified envelope. MF has antigen binding, serine-type endopeptidase inhibitor activity, and peptidase inhibitor activity. KEGG covered fat digestion and absorption, neuroactive ligand-receptor interaction, and the interaction between cytokine and Pancreatic secretion (**Figure 1E**).

**Figure 2:**
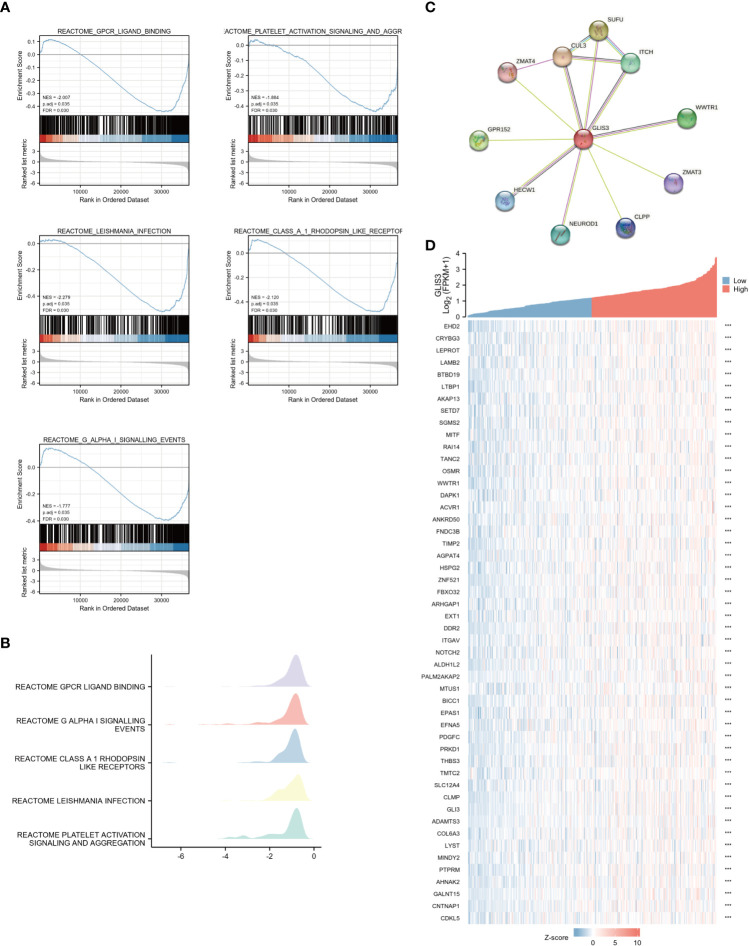
Enrichment analyses and PPI network **(A)** Enrichment analyses from GSEA. GLIS3 participates in five related pathways in gastric cancer: Reactome gpcr ligand binding, G alpha-I signaling events, class A 1 rhodopsin-like receptors, leishmania infection, and platelet activation signaling and aggregation. MSigDB was used for the gene set database. 1000 random sample permutations were performed. NES, normalized enrichment score; FDR, false discovery rate. **(B)** Mountain range map for GSEA enrichment analysis. **(C)** A PPI network consisting of 10 HUB genes. **(D)** Heat map showing the top 50 genes associated with GLIS3 co-expression in gastric cancer. (***P <0.001).

Using the MSigDB library, we performed GSEA analysis to identify better the biological processes associated with GLIS3. Reactome gpcr ligand binding, G alpha-I signaling events, class A 1 rhodopsin-like receptors, leishmania infection, and platelet activation signaling and aggregation exhibited significant differential enrichment among the significantly enriched gene collections (**Figures 2A, B**).

To find out the potential relationship between GLIS3 and other genes in gastric cancer, PPI network analysis was performed with the help of an online string (https://string-db.org/ ) database (**Figure 2C**). The 50 genes associated with GLIS3 with P>0.05 and the highest correlation were also represented using a single gene co-expression heat map (**Figure 2D**). In this case, CLU3 and SUFU are strongly associated with cancer progression, and recent studies suggest that they appear to be associated with iron death sensitivity (29–31)

### GLIS3 implies the proliferation and metastasis of gastric cancer cells

**Figure 3:**
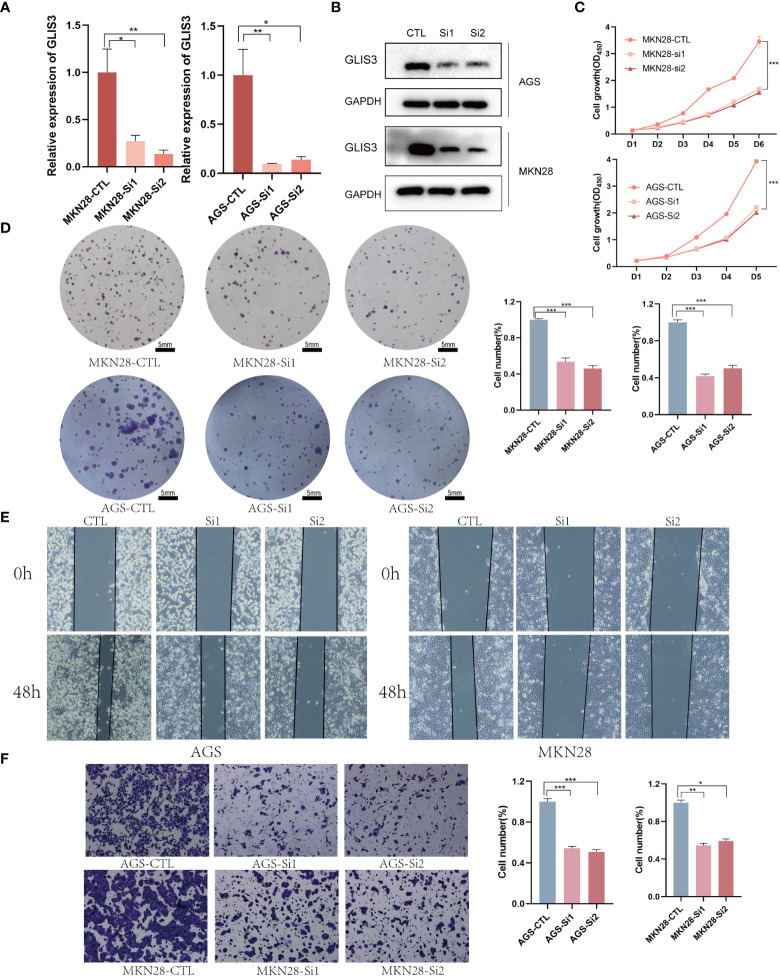
GLIS3 *in vitro* functional validation **(A)** Validation of GLIS3 knockdown efficiency in gastric cells by qRT-PCR assay. **(B)** Western Blot was used to analyze the silencing efficiency of gastric cancer cells. **(C)** The effect of GLIS3 knockdown on CCK-8 cell proliferation in MKN28 and AGS cells. **(D)** Plate cloning assay of the effect of GLIS3 knockdown on cell proliferation capacity in MKN28 and AGS cells. (Scale Bar=5mm) **(E)** GLIS3 was silenced in gastric cancer cells, and cell wound healing and microscopic observations were photographed at 0 and 48 h after scratching the AGS and MKN28cells surface. **(F)** Transwell assay detects the effect of GLIS3 silencing on the migratory ability of MKN28 and AGS cells. (***P <0.001, **P < 0.01, *P< 0.05).

To further test our hypothesis. The silencing of GLIS3 was achieved in gastric cancer cells AGS and MKN28 by transient transfection with Lipofectamine 3000 containing 1μg siRNA, and the transfection efficiency was detected by WB and qPCR after 48H collection of protein and RNA (**Figures 3A, B**). To verify whether GLIS3 affects the proliferative ability of gastric cancer, CCK8 and plate cloning experiments were performed using siRNA groups and a blank control (CTL) group. (**Figures 3A, D**) The results of the experiments showed that the proliferation ability of the cells was inhibited. Following that, transwells and cell scratch assay were used to detect the ability of gastric cancer cells to spread. The metastatic ability of gastric cancer cells was similarly inhibited after GLIS3 silencing, as indicated by the results (**Figures 3E, F**).

### Correlation between immune infiltration of GC and GLIS3 expression

**Figure 4:**
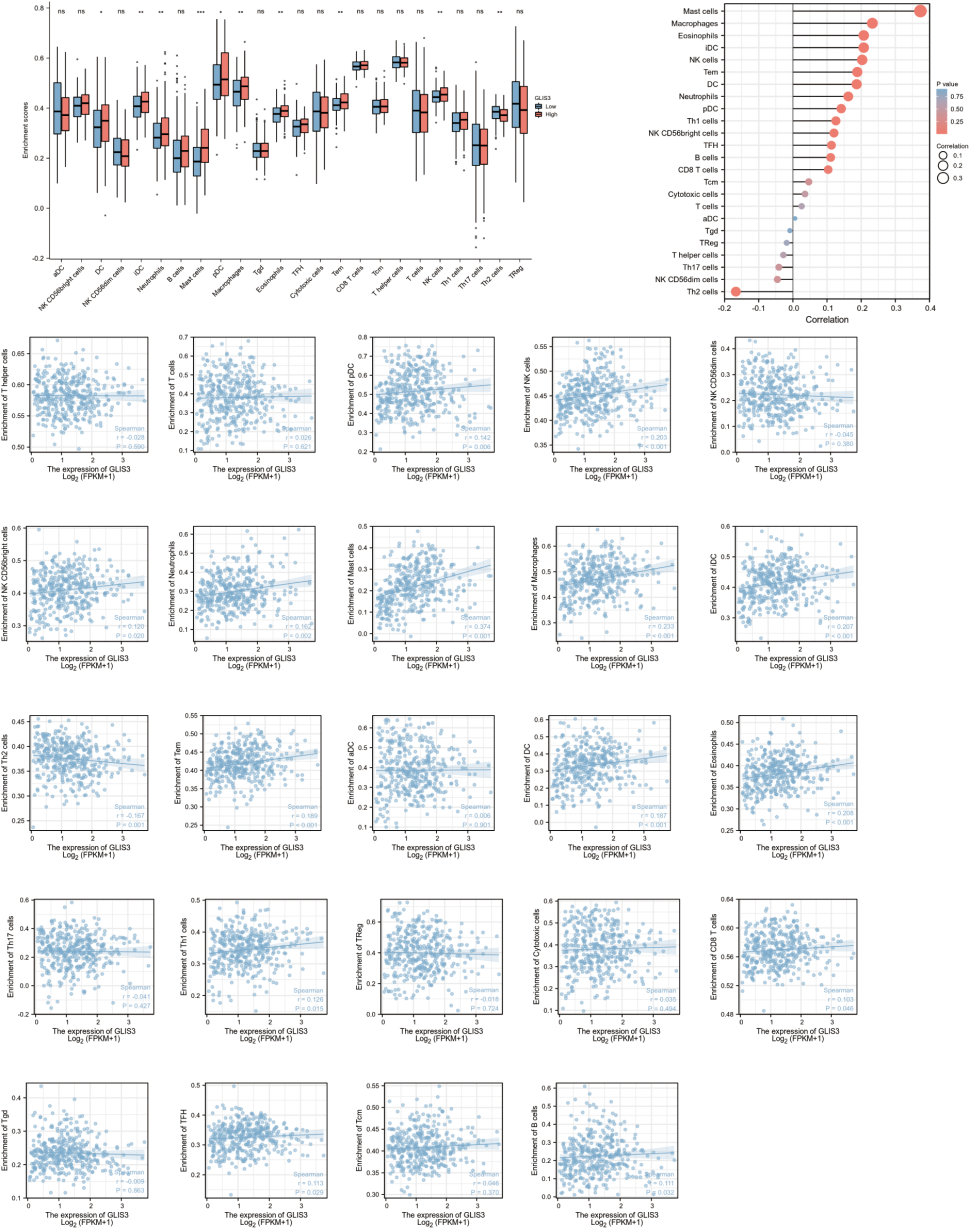
GLIS3 expression in STAD in relation to immune infiltration. **(A)** Expression of GLIS3 in gastric cancer is closely associated with immune cell infiltration. **(B)** Correlation of GLIS3 expression with 24 immune cells in gastric cancer. **(C)** Correlation of GLIS3 expression with the degree of infiltration of 24 immune cells and specific p-values in gastric cancer. (**P < 0.01, *P< 0.05, ns, No sense).

Tumor immune infiltration plays an important role in predicting OS incidence. the proportion of 24 immune cell subtypes in different GLIS3 expression groups showed that Mast cells (P<0.001), NK CD56 bright cells (P=0.069), TFH (T follicular helper, P=0.133), Th1 cells (P=0.288), pDCs (plasmacytoid dendritic cells, P<0.05), Eosinophils (P< 0.005), iDCs (immature DCs, P< 0.005), Macrophages (P< 0.005), Neutrophils (P=0.005), NK cells (P<0.005), Tcm (Central Memory T cell, P=0.224), CD8 T cells(P=0.343), Tem (Effective Memory T Cell, P<0.005), B cells (P=0.208), and DC (dendritic cell, P<0. 05), were significantly increased in high GLIS3 group, while aDCs (activated DCs, P=0.288), Treg (regulatory T cells) (P=0.221), T cells (P=0.924), NK CD56 dim cells (P=0.090), Cytotoxic cells (P=0.936), Tgd (T gamma delta, P=0.936), T helper cells (P=0.804), Th17 cells (P=0.662), and Th2 cells (P=0.005) were significantly decreased (**Figures 4A, B**).

Furthermore, the expression of GLIS3 was associated with Treg (regulatory T cells, r=− 0.018, P =0.724), NK CD56 dim cells (r=−0.045, P=0.380), Tgd (T gamma delta, r =− 0.009, P=0.863), T helper cells (r=− 0.028, P=0.590), Th17 cells (r= − 0.041, P=0.427), and Th2 cells (r=− 0.167, P= 0.001) shown negative correlation. A positive correlation was found between GLIS3 expression and infiltration levels of aDCs (activated DCs, r=0.006, P=0.901), B cells (r=0.111, P<0.05), CD8 T cells (r = 0.103, P <0.05), Cytotoxic cells (r = 0.035, P =0.494), DC (dendritic cell, r=0.187, P<0.001), Eosinophils (r=0.208, P<0.001), iDCs (immature DCs, r=0.207, P<0.001), Macrophages (r = 0.233, P <0.001), Mast cells (r =0.374, P<0.001), Neutrophils (r=0.162, P<0.005), NK CD56 bright cells (r= 0.120, P<0.05), NK cells (r=0.203, P<0.001), pDCs (plasmacytoid dendritic cells, r=0.142, P<0.05), T cells (r=0.026, P=0.621), Tem (Effective Memory T Cell, r=0.189, P<0.001), Tcm (Central Memory T cell, r=0.046, P=0.370), TFH (T follicular helper, r=0.113, P=0.029), and Th1 cells (r= 0.126, P<0.05) (**Figure 4C**).

### Expression of GLIS3 is associated with immune checkpoints

**Figure 5:**
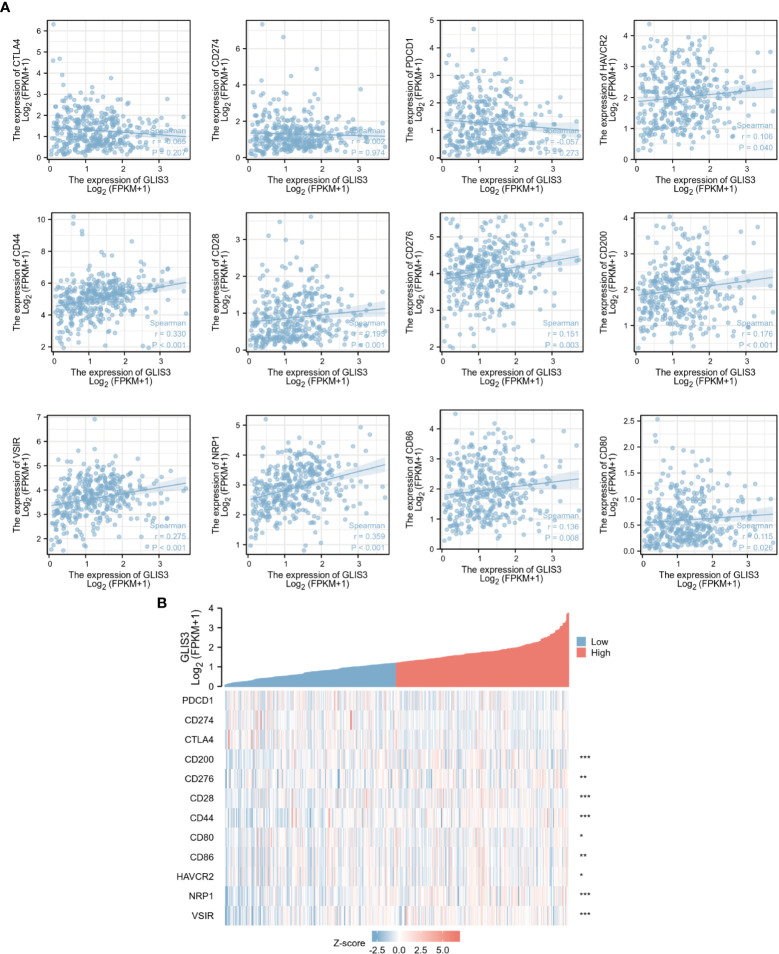
Correlation of GLIS3 expression with immune checkpoints. **(A)** Correlation between GLIS3 expression in GC and immune checkpoints (PD1, PD-L1, CTLA4, CD200, CD276, CD28, CD44, CD80, CD86, HAVCR2, NRP1, and VSIR) **(B)** Heat map depicting immune checkpoints correlated with GLIS3 expression in TCGA-STAD. (*** P <0.001, **P < 0.01, * P< 0.05).

Immune checkpoints are a series of molecules expressed on immune cells and regulate the degree of immune activation. Tumor cells express substances that activate immune checkpoints, blocking the antigen presentation process in tumor immunity, suppressing immune function and causing immune escape. In relation, the expression of GLIS3 in connection to immunological checkpoints such as PD1, PD-L1, CTLA4, CD200, CD276, CD28, CD44, CD80, CD86, HAVCR2, NRP1, and VSIR was studied. GLIS3 is positively correlated with many immune checkpoints. Among them, CD200, CD28, CD44, NRP1, and VSIR had a robust correlation (P<0.001).CD276, CD80, CD86 and HAVCR2 expression levels were favorably linked with GLIS3 expression (P<0.05, **Figures 5A, B**). These results suggest that GLIS3 is intimately involved in regulating immune interactions and may regulate tumor immune escape.

### Clinical characteristics and prognosis analysis related to GLIS3 expression

From the TCGA data portal in October 2022, 375 patients with the required clinical features were extracted. **Table 1** lists the detailed clinical features. Among 375 subjects, 188 demonstrated high GLIS3 expression, and 187 demonstrated low expression. There were 134 men and 241 women present. The average age of the participants was 65. The mean age of all participants was 65 years. Stage STAD: 53 patients in stage I, 111 in stage II, 150 in stage III, and 38 in stage IV. GLIS3 expression was connected with regional lymph node condition, PFI event, DSS event, and Anatomic neoplasm subdivision (**Table 1**)

**Table 1:** Clinical features associated with high or low expression of GLIS3 in patients with gastric cancer.

Characteristic	Low expression of GLIS3	High expression of GLIS3	p
n	187	188	
N stage, n (%)			0.008
N0	63 (17.6%)	48 (13.4%)	
N1	52 (14.6%)	45 (12.6%)	
N2	39 (10.9%)	36 (10.1%)	
N3	24 (6.7%)	50 (14%)	
PFI event, n (%)			0.007
Alive	138 (36.8%)	113 (30.1%)	
Dead	49 (13.1%)	75 (20%)	
DSS event, n (%)			0.045
Alive	141 (39.8%)	122 (34.5%)	
Dead	37 (10.5%)	54 (15.3%)	
Anatomic neoplasm subdivision, n (%)			0.049
Antrum/Distal	69 (19.1%)	69 (19.1%)	
Cardia/Proximal	22 (6.1%)	26 (7.2%)	
Fundus/Body	71 (19.7%)	59 (16.3%)	
Gastroesophageal Junction	14 (3.9%)	27 (7.5%)	
Other	4 (1.1%)	0 (0%)	

**Figure 6:**
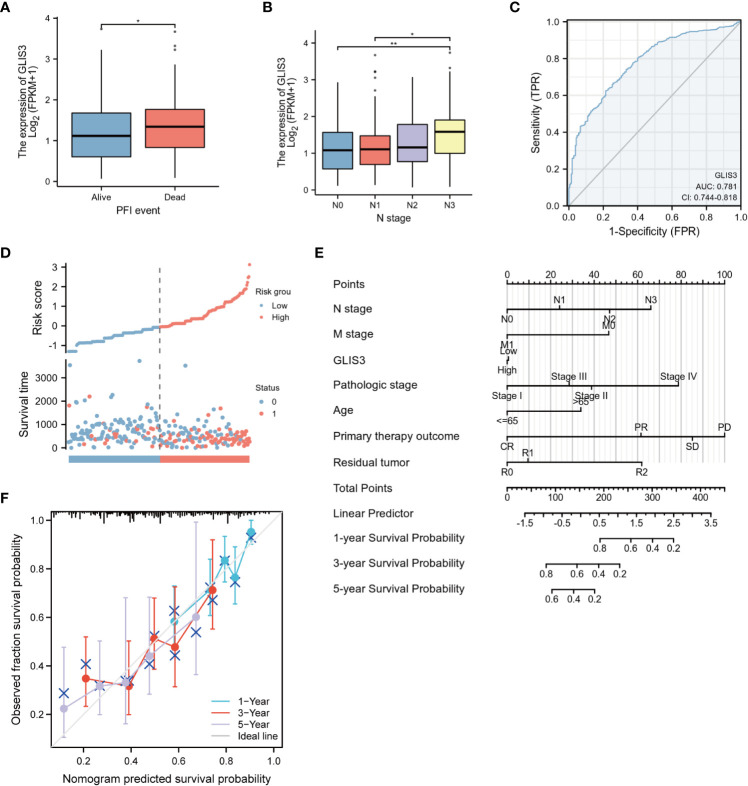
Diagnostic value of GLIS3 expression for STAD. Relationship between GLIS3 expression and clinicopathological features of STAD and diagnostic value. **(A)** PFI Event. **(B)** N Stage. **(C)** ROC analysis of GLIS3 showed that GLIS3 has the ability to differentiate between tumor and normal tissue. **(D)** Expression, risk score and survival time distribution of GLIS3. **(E)** Nomogram for predicting 1-year, 3-year and 5-year OS probabilities in patients with gastric cancer. **(F)** Calibration curve model to validate the predictive value of OS prediction for 1- year, 3-year and 5-year survival (**P < 0.01, *P< 0.05).

Analyzing the primary clinical characteristics of the low and high GLIS3 expression groups, GLIS3 expression was higher in the PFI event death group, and expression increased with increasing staging in the N stage. (**Figures 6A, B**). Based on GLIS3 gene expression data, ROC curve analysis was done to determine the diagnostic utility of this gene. With a measurement of 0.781, the area has a high diagnostic value. (**Figure 6C**).

Univariate analysis revealed that age, TMN classification, pathologic stage, Primary therapeutic outcome, and Residual tumor are linked with GLIS3 expression level and OS (P<0.05). In addition, these risk factors were included in multivariate COX regression models (**Table 2**). The association between risk score, survival time, and the GLIS3 expression profile was then investigated. (**Figure 6D**) Clinical characteristics were incorporated into the nomogram model, and the anticipated probabilities of the calibration curve were congruent with the observed data. (**Figures 6E, F**).

**Table 2:** Univariate and multifactorial COX regression analysis of clinical characteristics in relation to overall survival.

Characteristics	Total(N)	Univariate analysis		Multivariate analysis	
		Hazard ratio (95% CI)	P value	Hazard ratio (95% CI)	P value
T stage	362				
T1	18	Reference			
T2	78	6.725 (0.913-49.524)	0.061	27439473.697 (0.000-Inf)	0.996
T3	167	9.548 (1.326-68.748)	**0.025**	29401820.042 (0.000-Inf)	0.996
T4	99	9.634 (1.323-70.151)	**0.025**	32118936.113 (0.000-Inf)	0.995
N stage	352				
N0	107	Reference			
N1	97	1.629 (1.001-2.649)	**0.049**	1.792 (0.718-4.471)	0.211
N2	74	1.655 (0.979-2.797)	0.060	2.332 (0.761-7.145)	0.138
N3	74	2.709 (1.669-4.396)	**<0.001**	2.892 (0.946-8.844)	0.063
M stage	352				
M0	327	Reference			
M1	25	2.254 (1.295-3.924)	**0.004**	0.515 (0.137-1.935)	0.326
GLIS3	370				
Low	185	Reference			
High	185	1.478 (1.062-2.055)	**0.020**	1.070 (0.687-1.666)	0.766
Pathologic stage	347				
Stage I	50	Reference			
Stage II	110	1.551 (0.782-3.078)	0.209	1.058 (0.286-3.916)	0.932
Stage III	149	2.381 (1.256-4.515)	**0.008**	0.901 (0.158-5.141)	0.907
Stage IV	38	3.991 (1.944-8.192)	**<0.001**	1.789 (0.256-12.490)	0.557
Age	367				
<=65	163	Reference			
>65	204	1.620 (1.154-2.276)	**0.005**	1.656 (1.052-2.606)	**0.029**
Primary therapy outcome	313				
PD	64	Reference			
SD	16	0.590 (0.267-1.305)	0.193	1.016 (0.396-2.605)	0.974
PR	4	0.750 (0.233-2.412)	0.629	0.654 (0.150-2.861)	0.573
CR	229	0.215 (0.145-0.319)	**<0.001**	0.279 (0.169-0.461)	**<0.001**
Residual tumor	325				
R0	294	Reference			
R1	15	1.910 (0.961-3.797)	0.065	1.214 (0.508-2.901)	0.662
R2	16	7.866 (4.325-14.304)	**<0.001**	2.111 (0.645-6.906)	0.217
Histological type	369				
Mucinous Type	19	Reference			
Diffuse Type	63	3.474 (1.048-11.515)	**0.042**	2.657 (0.579-12.203)	0.209
Signet Ring Type	11	8.442 (2.234-31.893)	**0.002**	2.949 (0.513-16.944)	0.225
Not Otherwise Specified	202	4.095 (1.291-12.987)	**0.017**	3.297 (0.764-14.228)	0.110
Papillary Type	5	5.925 (1.193-29.429)	**0.030**	9.728 (1.431-66.124)	**0.020**
Tubular Type	69	3.310 (1.000-10.956)	0.050	1.886 (0.415-8.571)	0.411

Bold values denote statistical significance at the P < 0.05 level.

**Figure 7:**
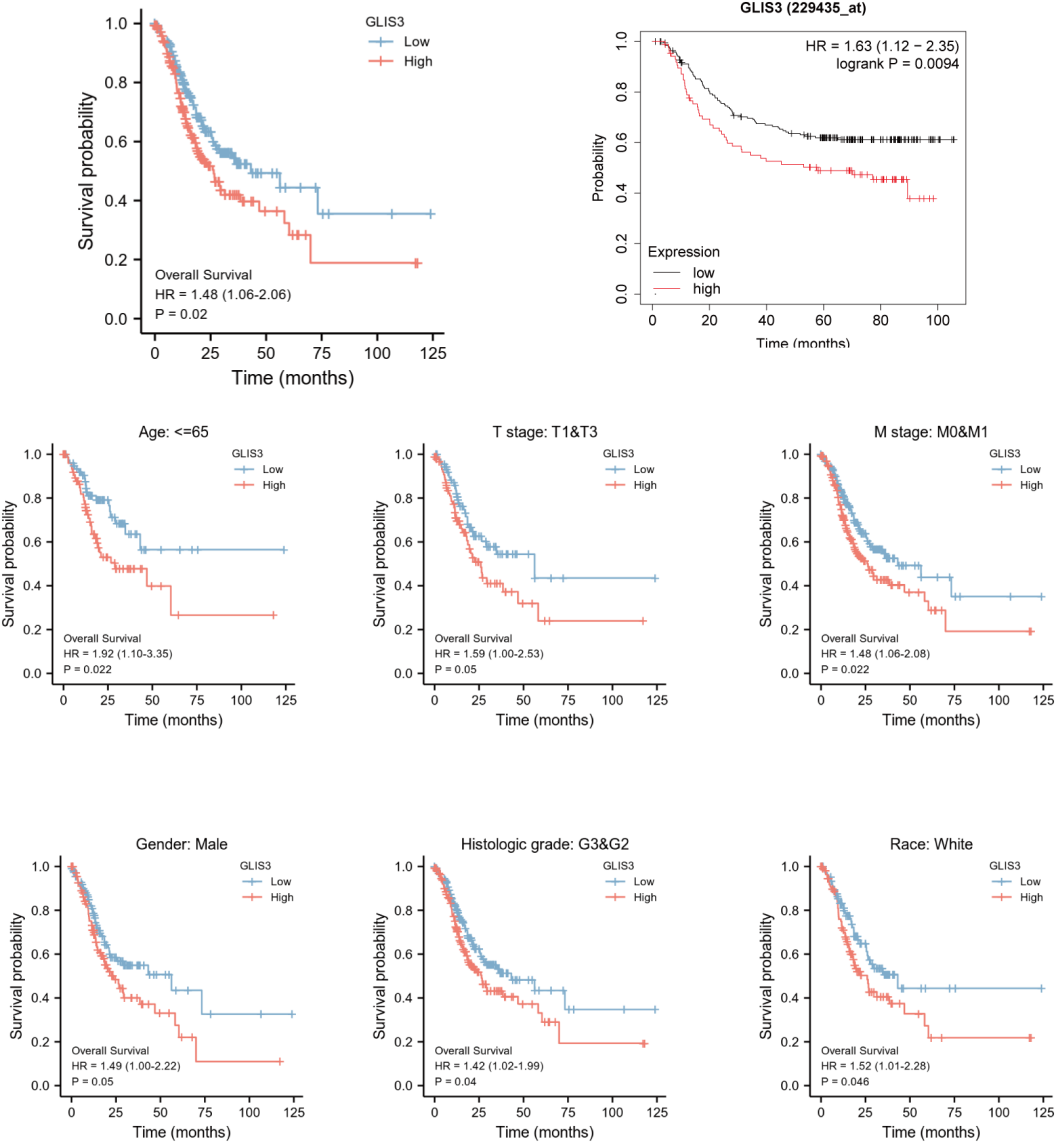
Kaplan-Meier curve for overall survival in gastric cancer. **(A)** High levels of GLIS3 expression in TCGA database and GEO database often correlate with poor prognosis (OS) in GC patients. **(B)** Kaplan-Meier prognostic analysis of Age≦65, Stage T1&T3, Stage M0&M1, Male, Histological Grade, White Ethnicity Regarding GLIS3 high and low expression scores in GC.

Kaplan-Meier survival analysis was performed using the TCGA database and GEO database GSE62254. Poor prognosis in patients with high GLIS3 expression. The results after performing subgroup analysis showed that poor prognosis in patients with high GLIS3 expression was associated with T stage (P<0.05), M stage (P<0.05), Age (P<0.05), male (P<0.05), race-white (P<0.05) and Histologic grade (P<0.05), respectively (**Figure 7**).

## Discussion

Gastric cancer is the fourth highest cause of cancer-related death (1). Gastric cancer begins in the innermost layer of the stomach, infiltrates more profound into the stomach wall, and spreads to nearby lymph nodes, the liver, the lung, and the peritoneum. Since early stomach cancer is typically asymptomatic, many individuals are discovered with the disease at an advanced stage. Surgical resection may be able to cure early-stage, locally-confined stomach cancer. Advanced tumors can only be treated with palliative care and have a poor prognosis. Exploring the genetic processes of gastric carcinogenesis and prognostic markers may lead to developing more effective treatments for people with gastric cancer.

The mouse GLIS3 gene with five C2H2-type zinc finger motif highly similar to the Gli and Zic gene families was found for the first time in 2003 (14). GLIS3 possesses DNA-binding transcription factor and DNA-binding transcription activator activity, RNA polymerase II specificity, and is implicated in the formation of pancreatic -cells and the thyroid (18). The possible prognostic impact of GLIS3 in gastric cancer has not been reported. Our data indicate that the expression of GLIS3 is substantially linked with immune infiltration and OS in patients with GC. We examined the relationship between GLIS3 and immune cells, which suggests that GLIS3 may be associated with immune infiltration. As the tumor microenvironment has been explored, Immune cells play a complex and crucial role in tumor growth (32).

We found that GLIS3 expression positively correlated with most immune cells. In tumors with high GLIS3 expression, immune cells were highly infiltrating. And GLIS3 tended to show increased expression in gastric cancer. The tumor microenvironment (TME) is conducive to the growth and expansion of cancer cells. Many cell types are involved in the TME and host anti-tumor immune responses, and normal tissue destruction also occur in the TME (33–35). This may be why increased GLIS3 expression promotes gastric carcinogenesis and a bad prognosis: disruption of the TME in concert with immunosuppressive cells results in immune evasion. Among immune cells, macrophages demonstrated a stronger connection with GLIS3 expression (P<0.001) M1-type macrophages can destroy tumor cells and protect against pathogen invasion, but M2-type macrophages primarily promote tumor growth, invasion, and metastasis. The majority of macrophages in tumor tissues have the phenotype and function of M2 macrophages, and their degree of infiltration is adverse effect (36–38). Due to the phenotypic alteration of tumor-associated macrophages, the immune milieu is shifted from an anti-tumor state to an immunosuppressive state, indicating an increased risk of tumor invasion. Mast cells are immune cells seen in human cancers present in all vertebrates and were named by Paul Ehrlich (39–41). Mast cell density is correlated with angiogenesis, the number of metastatic lymph nodes, and patient survival in gastric cancer. Mast cells promote the development of gastric cancer by releasing angiogenic (VEGF-A, CXCL8, MMP-9) and lymphangiogenic components (VEGF-C, VEGF-F) (41–45). And in our immune infiltration analysis, mast cells were the immune cells with the most significant positive correlation with GLIS3, suggesting a higher infiltration rate of mast cells in tumors, leading to dysregulation of antitumor effects and correlating with poor patient prognosis (46). Also, NK cells, which are highly associated with GLIS3 expression, impact on immunotherapy, and targeting NK cells may improve anti-tumor immune responses (47).

Furthermore, we discovered a clear correlation between GLIS3 expression and immunological checkpoints such as NRP1, CD200, and CD276. Research by Dario A.A. Vignali’s team suggests that blocking NRP1, a potential immune checkpoint in T cells, could improve immunotherapy and help prevent cancer recurrence (48). And CD200 (OX-2), on the other hand, is a cell surface glycoprotein that confers immune escape by suppressing the alloimmune and autoimmune responses through its receptor CD200R (49).B7-H3 (CD276) is overexpressed in a variety of tumor types. It is a promising target for anticancer immunotherapy. In addition to its immunomodulatory effects, B7-H3 has intrinsic tumorigenic activities that enhance cell proliferation, migration, invasion, angiogenesis, metastasis, and anti-tumor drug resistance (50). From this, we can prove that GLIS3 may alter tumor immunology and may be a potential immunotherapy treatment target, instead of a simple prognostic biomarker. In terms of prognosis, in the group with high GLIS3 expression, the chance of survival was lower for T stage, M stage, age, male, white race, and histologic grade, indicating that GLIS3 has some predictive effect on prognosis.

To predict 1- years, 3- years, and 5-years OS in GC, we built a prognostic nomogram model of GLIS3 expression levels based on COX regression analysis. Time-dependent ROC curves demonstrate the nomogram’s dependable prediction capabilities. Our model may give a new starting point for prognostic prediction and individualized assessment of patients with GC. Nonetheless, this study still has certain drawbacks. The regulatory mechanisms and signaling pathways linked with GLIS3 require additional analysis. Future multicenter research should be conducted to validate the predictive model.

## Conclusion

GLIS3 is significantly expressed in GC, and high expression is related to a bad prognosis. Our research indicates that GLIS3 is a potential prognostic factor and genetic therapeutic target. Future research will concentrate on the mechanism of action of GLIS3 in GC so that GLIS3 can become a therapeutic and prognostic factor for the benefit of patients.

## Data availability statement

Publicly available datasets were analyzed in this study. This data can be found here: https://xenabrowser.net/datapages/ https://cancergenome.nih.gov https://www.ncbi.nlm.nih.gov/geo.

## Ethics statement

The studies involving human participants were reviewed and approved by the Ethics Committee of the First Affiliated Hospital of Zhengzhou University. Written informed consent for participation was not required for this study in accordance with the national legislation and the institutional requirements.

## Author contributions

YQ, YD, and ZW conceived and designed the experiments. CW and ZW performed functional enrichment analyses. DL and ZW completed the supplemental experiment. YD and CC analyzed the results and wrote the manuscript. The manuscript was written through the contributions of all authors. All authors contributed to the article and approved the submitted version.
